# Fatigue in patients with advanced renal cell carcinoma receiving sunitinib on an intermittent versus continuous dosing schedule in a randomized phase II trial

**DOI:** 10.1002/cam4.286

**Published:** 2014-07-10

**Authors:** David Cella, Sally E Jensen, Elizabeth A Hahn, Jennifer L Beaumont, Beata Korytowsky, Helen Bhattacharyya, Robert Motzer

**Affiliations:** 1 Department of Medical Social Sciences, Northwestern University Feinberg School of MedicineChicago, Illinois; 2 Robert H. Lurie Comprehensive Cancer Center, Northwestern UniversityChicago, Illinois; 3 Division of Organ Transplantation, Department of Surgery, Northwestern University Feinberg School of MedicineChicago, Illinois; 4 Global Health Economics and Outcomes Research, Pfizer OncologyNew York City, New York; 5 Statistics, Pfizer Specialty Care BUNew York City, New York; 6 Memorial Sloan-Kettering Cancer CenterNew York City, New York

**Keywords:** Fatigue, quality of life, renal cell carcinoma, sunitinib

## Abstract

A phase II trial in advanced renal cell carcinoma (RCC) found no benefit in efficacy or safety between patients receiving oral sunitinib 50 mg/day for 4 weeks followed by 2-week off-treatment (Schedule 4/2) and those receiving 37.5 mg continuous daily sunitinib. We hypothesized that fatigue would have a more variable “on-off” effect with the 4/2 schedule. A total of 292 patients completed two fatigue-related items on Days 1 and 29 of each treatment cycle. Mean absolute slopes were compared across treatments. A planned analysis of item “I feel fatigued” demonstrated that the mean absolute slope was greater in Schedule 4/2 compared to continuous dosing (0.042 vs. 0.032, *P* = 0.003), and analysis based on the change from Day 1 to Day 29 (0.52 vs. 0.21, *P* = 0.002) and, separately, Day 29 to the next Day 1 (−0.38 vs. −0.05, *P* < 0.001) showed the changes to be significantly larger in Schedule 4/2 than continuous dosing. “I have a lack of energy” showed a similar pattern graphically, however, the planned analysis was not statistically significant based on the absolute slopes but was when Day 1 to Day 29 and Day 29 to Day 1 changes were analyzed separately. The 4/2 arm was associated with a greater degree of variability in fatigue reflecting a possible “on-off” effect whereby patients receiving the 4/2 schedule reported less fatigue at the beginning of each cycle compared to Day 29. The findings can inform care for individuals with advanced RCC receiving intermittent dosing of sunitinib.

## Introduction

The incidence of kidney cancer continues to rise, with renal cell carcinoma (RCC) comprising the majority of kidney cancer diagnoses 1. Approximately 20–30% of individuals with RCC are initially diagnosed with metastatic disease 2 and among those diagnosed with early stage cancer, 23% will progress to metastatic disease 3,4. Historically, conventional chemotherapy and immunotherapy with cytokines, interferon (IFN-*α*), and interleukin (IL-2) produced limited results in advanced RCC. However, the emergence of targeted therapies, such as vascular endothelial growth factor (VEGF) tyrosine kinase inhibitors (TKIs) and mammalian targets of rapamycin (mTORs), offers promise for improved clinical outcomes in advanced RCC. Sunitinib malate (SUTENT^©^; Pfizer, Inc., New York, NY) is an oral VEGF TKI approved as first-line treatment for advanced RCC 5, which has demonstrated superior clinical and quality of life (QOL) outcomes compared to IFN-*α* 6,7.

Although targeted therapies, such as sunitinib, confer better clinical and QOL outcomes than the traditional immunotherapies, toxicities related to targeted therapies may also affect QOL. For example, a recent randomized trial reported that adverse events resulted in dose reduction in 32% of patients receiving sunitinib 8. In this phase III registration study of sunitinib, fatigue was a commonly reported adverse event. Fatigue is a persistent, distressing, subjective sense of tiredness related to cancer treatment, not in proportion to recent activity, which interferes with typical functioning 12. Although the specific factors contributing to elevated fatigue among individuals receiving sunitinib remain unclear, the potential impact of fatigue on the QOL of patients may result in dose reductions, treatment interruptions, or treatment discontinuation.

A recent randomized phase II trial found no statistical benefit in time to progression, overall survival, or adverse events between sunitinib on Schedule 4/2 compared to a continuous daily dose of 37.5 mg 9. Furthermore, this randomized trial showed no significant differences between the two treatment arms in patient-reported QOL. However, there is reason to believe that, relative to patients who take a continuous daily dose, those assigned to the 4/2 treatment might experience an intermittent, on-again–off-again adverse event experience of symptoms such as fatigue. Here, we report the findings from a planned analysis that examined intracycle rate of change in fatigue by comparing the 4/2 treatment schedule to patients assigned to the continuous dose arm.

## Methods

### Study design

A detailed description of the study design has been previously reported 9. Briefly, in this multisite phase II trial, patients were randomized to receive oral sunitinib at a dose of either 50 mg/day on Schedule 4/2 (4-week on-treatment; 2-week off-treatment) or 37.5 mg/day on a continuous daily dosing schedule. Treatment was continued for up to 2 years or until disease progression, significant toxicity, or withdrawal of consent. Modifications in dose, secondary to individual patient toxicity, were permitted. This study was conducted in accordance with the International Conference on Harmonization Good Clinical Practice guidelines and received Institutional Review Board approval.

Findings related to the primary outcome, time to tumor progression, have been previously reported 9. This report examines the secondary endpoint of patient-reported fatigue.

### Patients

Patients aged ≥18 years with locally recurrent or metastatic RCC of clear cell type or with a clear cell component were eligible to participate in this study. Full eligibility criteria have been previously described 9. Patients were excluded if they had brain metastases, clinically significant cardiovascular events/disease during the past 6 months or uncontrolled hypertension. All patients provided written informed consent.

### Patient-reported outcomes assessment

Patient self-report questionnaires assessed patient-reported outcomes on Day 1 (a baseline assessment was taken in the first cycle prior to any treatment administration) and Day 29 of each treatment cycle, and at the end of treatment or patient withdrawal. The Functional Assessment of Cancer Therapy-Kidney Symptom Index-Disease-Related Symptoms subscale (FKSI-DRS 13,14) is a nine-item scale that measures symptoms related to kidney cancer, including lack of energy, fatigue, pain, bone pain, weight loss, shortness of breath, cough, fever, and hematuria. The FKSI-DRS was selected a priori as the primary patient-reported outcome endpoint. The present report describes the findings for the two FKSI-DRS items that assess aspects of fatigue: “I feel fatigued,” and “I have a lack of energy.” These two items were selected a priori for analysis in order to examine our hypothesis that the fatigue pattern would differ by sunitinib dosing schedule. Previous evidence supports the extraction of individual questions given their relevance to a priori hypotheses 15–17.

### Statistical analysis

Patient-reported outcomes analyses were performed for the intention-to-treat population. Scores on the two FKSI-DRS fatigue items were summarized using means and medians at each assessment time point. The average of the absolute values of all adjacent assessment slopes across the first six cycles of treatment was computed for the two fatigue items, with greater slopes indicative of greater rate of change (both better and worse) in scores from the start to the end of the treatment cycle. Independent samples *t*-tests were performed to assess for differences between the mean absolute slope for each treatment arm for the two FKSI-DRS items pertaining to fatigue.

Since the type of research question posed in this study is not associated with a typical statistical analysis strategy, alternative analysis techniques were considered. An additional analysis included the comparison of the two treatment arms on three derived scores: (1) the change from Day 1 of one cycle to Day 1 of the next cycle, (2) the change from Day 1 to Day 29 within cycles, and (3) the change from Day 29 to Day 1 of the next adjacent cycle. The average of each score was calculated for each patient and independent samples *t*-tests performed to assess for differences between the treatment arms.

Finally, to further explore the on-off effect of the 4/2 regimen, a one-sample paired comparison of the mean Day 1 values versus the mean Day 29 values was also carried out, with each patient being considered a pair.

## Results

### Patient characteristics

Between January 2007 and June 2008, 292 eligible patients were enrolled and randomly assigned to the Schedule 4/2 or continuous daily dosing treatment arm. At least two patient-reported outcomes assessments (e.g., baseline and at least one follow-up) were completed during the first six cycles of treatment by 91.1% (*N* = 133) and 90.4% (*N* = 132) of individuals assigned to the Schedule 4/2 and continuous daily dosing treatment arm, respectively.

### Fatigue rate of change

**Figure 1:**
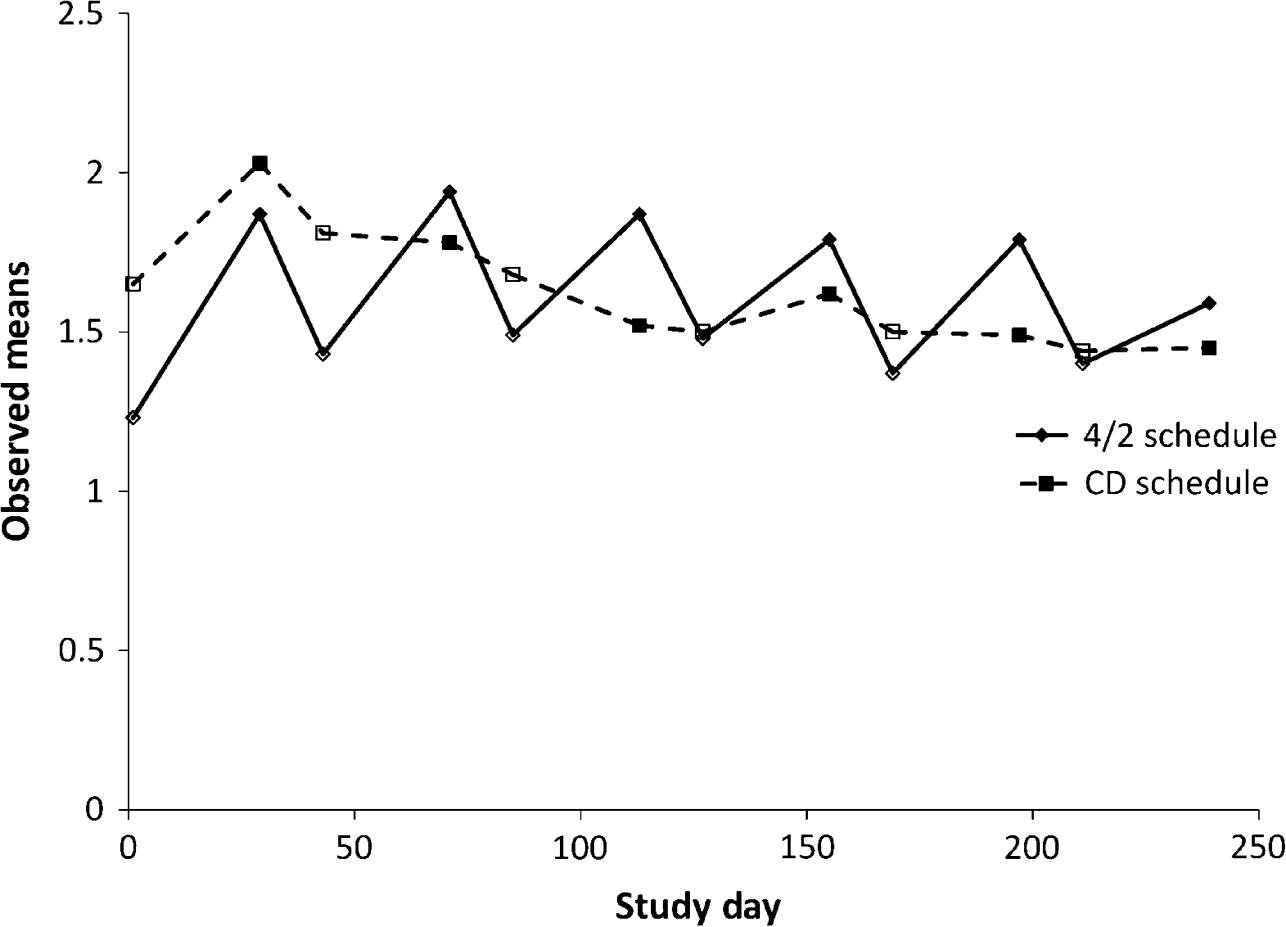
Observed mean scores on the FKSI-DRS item “I feel fatigued” from start to end of each treatment cycle for participants assigned to the Schedule 4/2 arm and to the continuous daily dosing arm. One treatment cycle is equivalent to 42 days. Open symbols denote Day 1 of each cycle. FKSI-DRS, Functional Assessment of Cancer Therapy-Kidney Symptom Index-Disease-Related Symptoms subscale.

Figure1 depicts the observed mean scores on the FKSI-DRS item “I feel fatigued” from start to end of each treatment cycle for participants assigned to Schedule 4/2 arm and to the continuous daily dosing arm. There was a significant difference in the baseline mean for the FKSI-DRS item “I feel fatigued,” between the Schedule 4/2 arm (*M* = 1.23, SD = 1.18) and the continuous daily dosing arm (*M* = 1.65, SD = 1.20), *P* < 0.01. For the FKSI-DRS item “I feel fatigued,” the mean absolute values of all adjacent assessment slopes in the Schedule 4/2 arm (*M* = 0.042, SD = 0.028) were significantly greater than the mean slopes in the continuous daily dosing arm (*M* = 0.032, SD = 0.026), *t*(263) = 2.99, *P* = 0.003. The follow-on analysis also provides supporting evidence for the hypothesis of different patterns. There was no significant difference between arms in change from Day 1 of one cycle to Day 1 of the next (mean difference = −0.02, 95% confidence interval = −0.18 to 0.13, *P* = 0.751), while there were significant differences seen in change from Day 1 to Day 29 within a cycle (mean difference = 0.31, 95% confidence interval = 0.11 to 0.51, *P* = 0.002) and change from Day 29 to Day 1 of the subsequent cycle (mean difference = −0.33, 95% confidence interval = −0.50 to −0.15, *P* < 0.001).

**Figure 2:**
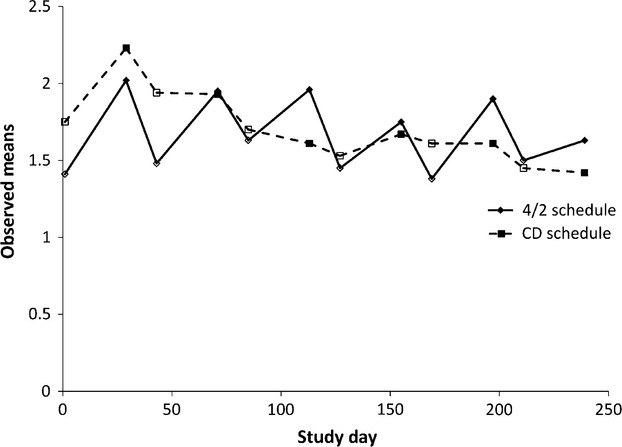
Observed mean scores on the FKSI-DRS item “I have a lack of energy” from start to end of each treatment cycle for participants assigned to the Schedule 4/2 arm and to the continuous daily dosing arm. One treatment cycle is equivalent to 42 days. Open symbols denote Day 1 of each cycle. FKSI-DRS, Functional Assessment of Cancer Therapy-Kidney Symptom Index-Disease-Related Symptoms subscale.

Figure2 depicts the observed mean scores on the FKSI-DRS item “I have a lack of energy” from start to end of each treatment cycle for participants assigned to the Schedule 4/2 arm and to the continuous daily dosing arm. There was a significant difference in the baseline mean for the FKSI-DRS item “I have a lack of energy,” between the Schedule 4/2 arm (*M* = 1.41, SD = 1.21) and the continuous daily dosing arm (*M* = 1.75, SD = 1.16), *P* < .05. For the item “I have a lack of energy,” the pattern was graphically similar but the differences in mean absolute values of all adjacent assessment slopes between the Schedule 4/2 arm (*M* = 0.037, SD = 0.026) and the continuous daily dosing arm (*M* = 0.036, SD = 0.028), *t*(263) = 0.48, were not significant, *P* = 0.629. The follow-on analysis, however, provided similar results to “I feel fatigued.” There was no significant difference between arms in change from Day 1 of one cycle to Day 1 of the next (mean difference = 0.01, 95% confidence interval = −0.14 to 0.15, *P* = 0.937), while there were significant differences in change from Day 1 to Day 29 within a cycle (mean difference = 0.31, 95% confidence interval = 0.12 to 0.49, *P* = 0.001) and change from Day 29 to Day 1 of the next cycle (mean difference = −0.26, 95% confidence interval = −0.44 to −0.09, *P* = 0.004).

For the paired comparison of Day 1 versus Day 29 in the 4/2 regimen, there was a statistically significant difference in fatigue (Day 1 mean 1.43, Day 29 mean 1.92; diff = 0.52, CI = 0.39–0.66, *P* < 0.001) as well as in lack of energy (Day 1 1.52, Day 29 2.05; diff = 0.54, CI = 0.42–0.67, *P* < 0.001).

## Discussion

Findings from a recent randomized phase II trial revealed no benefit in time to progression, overall survival, or adverse events between intermittent and continuous dosing schedules of sunitinib in advanced RCC 9. Although this trial also found no significant differences in QOL between the two treatment arms over the course of the trial, the findings demonstrated a pattern suggesting a greater rate of change in QOL within individual cycles among individuals assigned to the Schedule 4/2 arm. Our results based on absolute value analysis reveal that there was significantly greater rate of change in scores on the FKSI-DRS item “I feel fatigued” from the start to the end of a treatment cycle in the Schedule 4/2 arm as compared to the continuous dose arm. Moreover, graphical representation of scores on this item suggests the possibility of an “on-off” effect whereby participants in the Schedule 4/2 arm reported less fatigue at the beginning of each treatment cycle following the 2-week break, when compared to fatigue assessed at Day 29. This effect was supported by a follow-on analysis, where score changes were examined. For both fatigue and lack of energy, the magnitude of the on-off effect, relative to the continuous dose arm, was roughly 0.3 points on a 0–4 scale. Furthermore, in the examination of the Schedule 4/2 arm alone, the average change between Day 1 and Day 29 was ∼0.5 points for both items (SD = 0.7–0.8). Although this on-off pattern was observed in the data and is visibly evident in Figure1, numeric changes themselves were modest and Day 42 scores did not return to baseline levels during the early cycles. These differences were statistically significant, with *P* < 0.001; their meaning to patients is likely a matter of personal preference.

In the set of analyses carried out for the item “I feel lack of energy,” although similar results were obtained in the change analysis and the Day 1 versus Day 29 analysis as mentioned in the paragraph above, a significant difference based on the absolute value analysis was not obtained. Several plausible explanations might explain the difference between the fatigue and lack of energy results, noting that visually the results appear very similar. It is possible that a patient's endorsement of “fatigue” may not be synonymous with an endorsement of “lack of energy.” For example, while a lack of energy may be a common component of patient's experience or perception of fatigue, there is evidence to suggest that there are subtypes of fatigue 18 and the words patients use to describe their fatigue may reflect these different subtypes 19. Moreover, it is possible that patients' perception of fatigue encompasses symptoms beyond energy level, such as tiredness or weakness, which are often reported by individuals with advanced cancer 20.

Fatigue is a commonly associated toxicity which has been highlighted in clinical trials of sunitinib in mRCC 8. Among individuals with advanced cancer, fatigue is also the most frequently endorsed high priority symptom to monitor 21, and may adversely impact QOL via its effects on physical functioning, social functioning, activity level, and emotional well-being. The negative effects of fatigue on QOL may lead to dose reductions, treatment interruptions, nonadherence, and early treatment discontinuation, especially in the presence of other adverse events. In a recent meta-analysis comparing treatment-related fatigue, an adverse event associated with sunitinib, pazopanib, and sorafenib, Santoni et al. 22 report relative risks for all grade and high-grade fatigue. In RCC patients (2260 of 6996 total patients), when compared to pazopanib- and sorafenib-treated patients, sunitinib-treated patients had higher relative risk of all grade and high-grade fatigue. Given that the majority of these sunitinib-treated patients received the 4/2 schedule, our results may indicate that the patient experience of fatigue on the 4/2 sunitinib schedule might influence the report of fatigue as an adverse event. On the other hand, differences across these treatments in their effect upon cytokine levels and host immunity may also play a role 23,24. Further research is needed to understand this better.

The management of fatigue constitutes a critical aspect of treatment for individuals with advanced RCC receiving sunitinib. The goal for patients was to maximize their treatment benefit with an acceptable or manageable level of fatigue. With the efficacy and safety of sunitinib in its standard dose of 50 mg/day for 4 weeks on treatment followed by 2-week off-treatment (Schedule 4/2) already established, attention has turned as to whether different dosing paradigms, such as a lower dose without treatment breaks or shorter treatment and earlier breaks such as 50 mg/day for 2-week on-treatment followed by a week off treatment (Schedule 2/1), may improve tolerability of sunitinib for individuals with advanced RCC.

The identification of a significantly greater rate of change in fatigue from start to end of treatment cycle in individuals receiving sunitinib on an intermittent dosing schedule has important clinical implications. First, the findings of this study can be used to enhance education and preparedness among individuals with advanced RCC receiving sunitinib on Schedule 4/2. Increased patient education and preparedness may in turn improve patients' communication with oncology clinicians related to their experience of fatigue. Second, the discovery of a greater intracycle rate of change in fatigue highlights the time points within a treatment cycle that patients are most vulnerable to fatigue, which may improve clinicians' ability to effectively monitor fatigue and intervene on the factors that may contribute to it, such as thyroid abnormalities, anemia, or mood. Third, a better awareness of the greater intracycle rate of change in fatigue during intermittent dosing of sunitinib may also lead to improved implementation of fatigue-specific psychosocial and pharmacologic intervention. Additionally, the intermittent Schedule 4/2 dosing may benefit patients who maintain a specific schedule of activities and obligations and who need to know when they will not be on treatment, given that many of sunitinib's adverse events are reversible.

The results of this study highlight the importance of assessing fatigue in advanced RCC clinical trials, particularly when there are no differences between treatment arms in time to progression, overall survival, or adverse events. Recent trials comparing pazopanib and sunitinib highlight how fatigue and other patient-reported outcomes have emerged as important when weighing efficacy and safety 25,26. Although future work to improve fatigue monitoring and management in advanced RCC is warranted, the finding of greater rate of change in fatigue from start to end of treatment cycle in individuals receiving intermittently dosed sunitinib, but not in those receiving a continuous dose, can inform the clinical care of individuals with advanced RCC.
